# Case Report: Severe Osteoporosis and Preventive Therapy in RNA Polymerase III-Related Leukodystrophy

**DOI:** 10.3389/fneur.2021.622355

**Published:** 2021-02-26

**Authors:** Soma Furukawa, Misako Kunii, Hiroshi Doi, Naohide Kondo, Aya Ogura, Koichi Hirabuki, Takayuki Itoh, Naomichi Matsumoto, Fumiaki Tanaka, Masahisa Katsuno, Yasuhiro Ito

**Affiliations:** ^1^Department of Neurology, Toyota Memorial Hospital, Toyota, Japan; ^2^Department of Neurology, Nagoya University Graduate School of Medicine, Nagoya, Japan; ^3^Department of Neurology and Stroke Medicine, Yokohama City University Graduate School of Medicine, Yokohama, Japan; ^4^Wellness Promotion Center, Corporate Human Resource, Fuji Xerox Co., Ltd, Ebina, Japan; ^5^Hirabuki Clinic, Toyota, Japan; ^6^Faculty of Psychological and Physical Science, Aichi-Gakuin University, Nissin, Japan; ^7^Department of Human Genetics, Yokohama City University Graduate School of Medicine, Yokohama, Japan

**Keywords:** RNA polymerase III-related leukodystrophy, 4H leukodystrophy, POLR3A, bone fracture, osteoporosis, anti-RANKL monoclonal antibody, case report

## Abstract

RNA polymerase III (POLR3)-related leukodystrophy is an autosomal recessive form of leukodystrophy caused by homozygous or compound heterozygous mutations of the RNA polymerase III subunit genes, including subunit A (*POLR3A*). With respect to the manifestation triad, hypomyelination, hypodontia, and hypogonadotropic hypogonadism, it is also known as 4H leukodystrophy. Here, we report a 41-year-old woman of POLR3-related leukodystrophy by carrying compound heterozygous pathogenic variants of c.2554A>G (p.M852V) and c.2668G>T (p.V890F) in the *POLR3A* gene. She was amenorrheic and became a wheelchair user from the age of 15 years and suffered from multiple episodes of pathologic fractures, starting with a subtrochanteric fracture of the right femur after a tonic seizure at age 30 years. Head magnetic resonance imaging demonstrated hypomyelination and atrophies of the cerebellum, brainstem, and corpus callosum. Laboratory examination revealed a marked decrease of gonadotropins and estrogen, low bone density, and high bone resorption markers. Administration of anti-receptor activator of nuclear factor kappa-B ligand monoclonal antibody restored bone resorption markers to a normal level and prevented further pathological bone fractures. Our case emphasizes that osteoporosis should be recognized as a potential but serious complication in POLR3-related leukodystrophy. It may be feasible to prevent pathologic fractures by intensive osteoporosis therapy after endocrinological examinations and evaluation of bone metabolism.

## Introduction

RNA polymerase III (POLR3) consists of 17 subunits, including two major subunits, RPC1 and RPC2, encoded by the *POLR3A* and *3B* genes, respectively. Congenital hypomyelinating disorders caused by mutations of these genes are recently designated as POLR3-related leukodystrophy (1, 2). POLR3-related leukodystrophy exhibits autosomal recessive inheritance, and 4H leukodystrophy is a representative disorder characterized by the triad of hypomyelination, hypodontia, and hypogonadotropic hypogonadism (3).

Most patients show motor delay or regression in childhood, and many patients become non-ambulatory as a result of progressive spasticity and cerebellar ataxia. Cognitive decline is also common, and tooth agenesis, including hypodontia (lacking one to five permanent teeth) or oligodontia (lacking six or more permanent teeth), is a characteristic manifestation (4). A majority of female patients lack secondary sex characteristics and menstruation caused by hypogonadotropic hypogonadism (5). However, severe osteoporosis has not been emphasized in patients with POLR3-related leukodystrophy.

Here, we report a patient with POLR3-related leukodystrophy with repetitive pathologic bone fractures due to severe osteoporosis and discuss the mechanism and treatment strategy.

## Case Presentation

**Figure 1:**
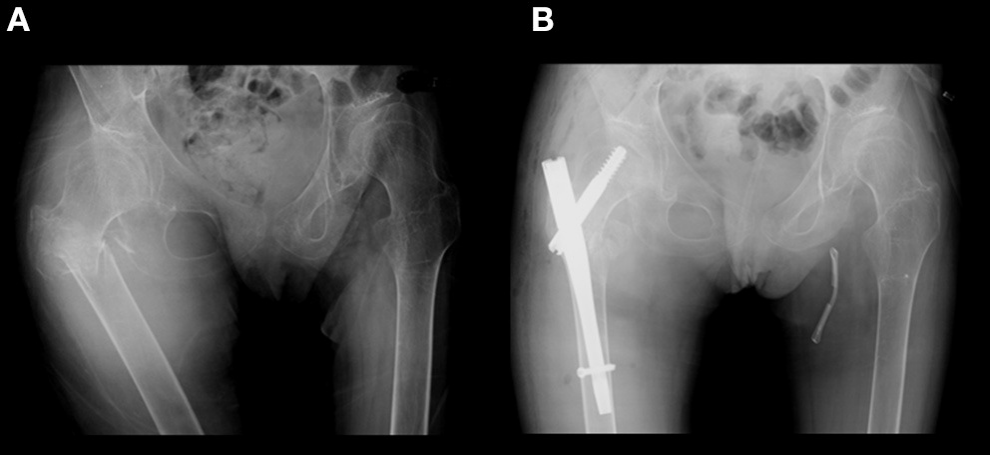
X-ray of her hip. **(A)** Subtrochanteric fracture of the right femur occurred after an episode of generalized tonic seizure. **(B)** Gamma nail osteosynthesis operation was performed.

The patient was a 41-year-old Japanese woman. She was healthy at birth, but walking was delayed. Her gait became slower during her elementary school years. Her parents noticed that most of her permanent teeth did not come in. Her secondary sex characteristics such as breasts and pubic hair were not developed. She had primary amenorrhea with no menarche in puberty. Due to her progressive gait disturbance along with cerebellar ataxia and limb spasticity, she became a wheelchair user at the age of 15 years. As her motor functions deteriorated, she became bedridden and required total assistance. She had recurrent generalized tonic seizures. Carbamazepine and phenytoin were administrated and increased in dosage up to 800 and 150 mg/day, respectively. She was referred to our department at age 27 years. She was hospitalized several times for aspiration pneumonia due to dysphagia, and prophylactic percutaneous endoscopic gastrostomy was performed. At age 30 years, she complained of pain and swelling of the right thigh after a long-lasting tonic attack without documented injury or other falls. X-ray examination revealed a subtrochanteric fracture of the right femur, and surgical treatment was performed (Figure 1). 4 months later, a fracture of the proximal phalanx of the left thumb occurred when she pressed her fingers against her abdomen during a tonic seizure. At age 34 years, she was admitted for further evaluation. On examination, her height was 162 cm and weight 35.4 kg. Neither development of mammary gland nor pubic hair were observed, corresponding to Tanner stage I. Oral examination confirmed oligodontia. Neurological findings included spontaneous horizontal nystagmus, difficulty in speaking and swallowing, and severe limb spasticity with an extension of both legs and flexion of both elbows and wrists. Tendon reflexes were markedly increased in upper and lower extremities without laterality. Jaw reflex and ankle clonus were manifest.

**Figure 2:**
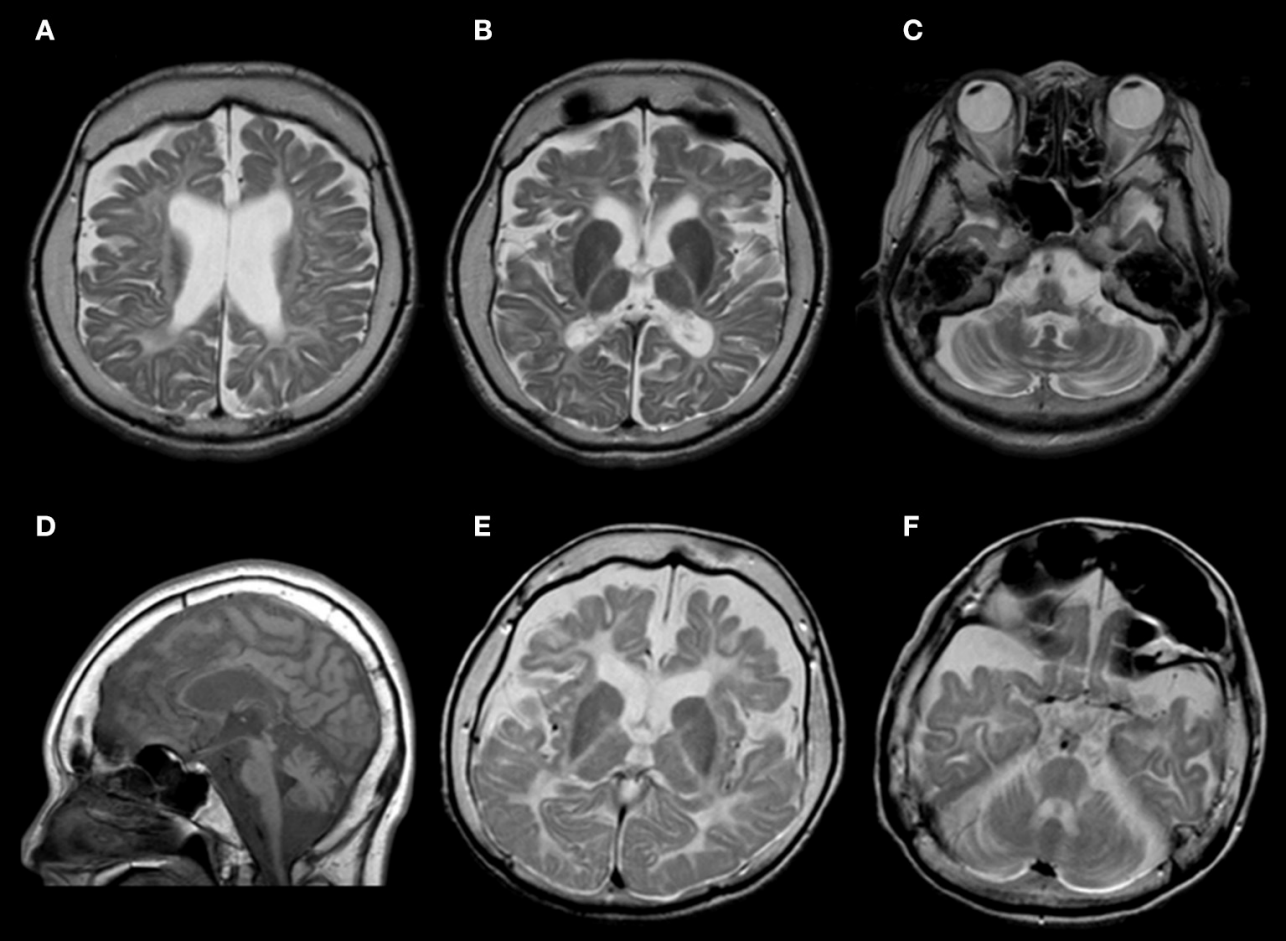
**(A–D)** Brain magnetic resonance imaging findings of this patient at age 34 years. **(A–C)** T2-weighted axial images revealed atrophy in the cerebrum and cerebellum. White matter is diffusely hyperintense, with relative preservation (T2 hypointensity) of the globus pallidus and cerebellar dentate nucleus. **(D)** T1-weighted sagittal images showed cerebellar atrophy, brainstem atrophy, and thinning of the corpus callosum. **(E,F)** Brain magnetic resonance imaging findings of her elder sister at age 30 years. T2-weighted axial images showed a similar pattern.

**Figure 3:**
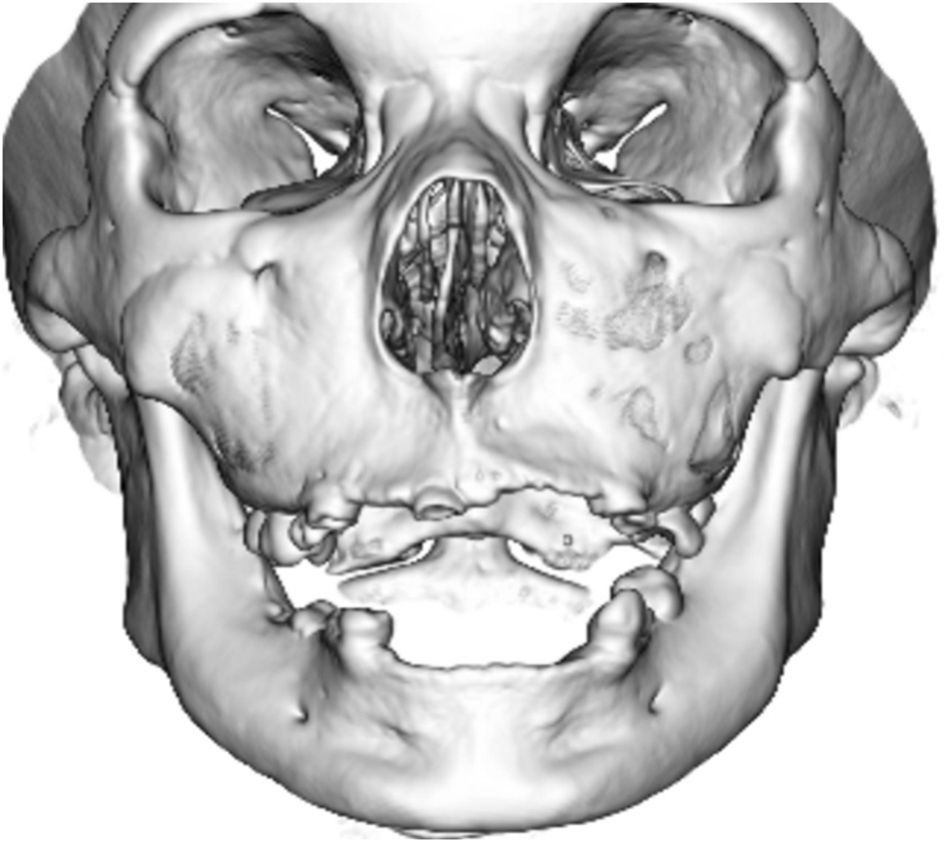
Computed tomographic image of her skull. Her primary teeth remained, and she had a congenital defect in her permanent teeth.

Neuroradiologically, head magnetic resonance imaging T2-weighted images showed brain atrophy and diffuse hyperintensity in the white matter, indicating hypomyelination. In contrast, myelination was relatively well-preserved in the globus pallidus, cerebellar dentate nucleus, and anterior lateral nucleus of the thalamus. Thinning of the corpus callosum and brain stem and cerebellar atrophy were also profound (Figures 2A–D). Skull computed tomography and dental examination revealed remaining deciduous teeth and a congenital defect in her permanent teeth (Figure 3).

**Figure 4:**
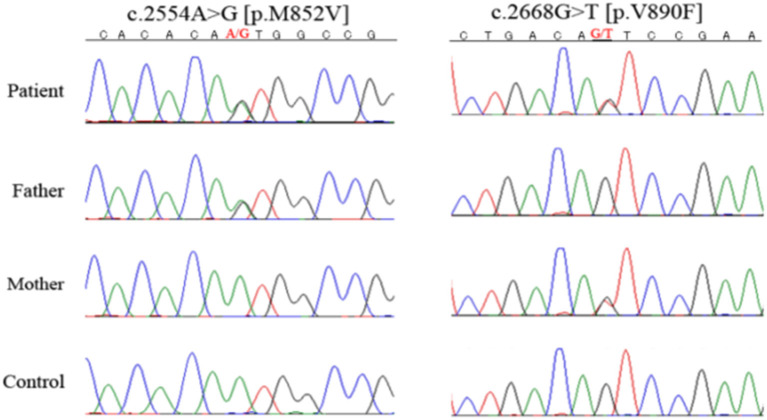
DNA sequencing. She had heterozygous mutations of c.2554A>G (p.M852V) and c.2668G>T (p.V890F) in the *POLR3A* gene. Her parents had different *POLR3A* gene mutations, with the father heterozygous for c.2554A>G (p.M852V) and the mother for c.2668G>T (p.V890F).

On endocrinological examination, luteinizing hormone, follicle-stimulating hormone, and estradiol were all below detectable levels, indicating hypogonadotropic hypogonadism. Prolactin level was 1.1 μg/L (1.2–40.9 μg/L). Serum thyroid hormones (TSH, FT3, and FT4), ACTH, cortisol, DHEA-S, GH, and IGF-1 were all within the normal range. Her karyotype was 46, XX. DNA sequencing using a custom-designed gene panel was performed with the surrogate consent of her guardians. Compound heterozygous mutations were identified at c.2554A>G (p.M852V) and c.2668G>T (p.V890F) in the *POLR3A* gene (6). C.2554A>G (p.M852V) was previously reported as a causative mutation in POLR3A-related leukodystrophy, but c.2668G>T (p.V890F) is a novel mutation, and no such mutation is reported in exome analysis of healthy individuals (Figure 4). To predict whether this novel mutation is deleterious or not, three *in silico* analyses were performed: Sorting Intolerant From Tolerant (SIFT), PolyPhen-2, and Mutation Taster. The results were “damaging” (score 0.00) on SIFT (http://sift.jcvi.org/), “probably damaging” (score 0.998) on PolyPhen-2 (http://genetics.bwh.harvard.edu/pph2/), and “disease causing” (score 1.00) on Mutation Taster (http://www.mutationtaster.org/). This mutation causes a substitution of an amino acid in RNA polymerase Rpb1, domain 5, which is conserved among various species, strongly suggesting it is deleterious. In conclusion, she was diagnosed with POLR3A-related leukodystrophy.

Both of her parents were healthy and had no consanguineous relationship. Her parents had different *POLR3A* gene mutations with paternal heterozygous of c.2554A>G (p.M852V) and maternal heterozygous of c.2668G>T (p.V890F). Her elder sister was healthy at birth, but limb spasticity and walking impairment appeared at age 2 years. Her development peaked at the age of 4 years and regressed after that. She had hypodontia and amenorrhea as well. She was confined to bed from age 12 years. Her head magnetic resonance imaging at age 30 years showed diffuse hyperintensity in the white matter and relatively retained myelin in the basal ganglia and thalamus, both of which were similar to those observed in her sibling (Figures 2E,F). She suffered from recurrent aspiration pneumonia with progressive respiratory failure, underwent a tracheotomy at age 30 years, and died at the age of 32 years. Genetic analysis was not performed. However, we strongly suspected she had the same disease.

We conducted further analysis of bone metabolism because the younger sister had repeated bone fractures. On the bone density testing, the T-score at L2–4 was exceptionally low at −4.9 SD. Bone metabolism index including Ca, P, ALP, PTH-intact, and 1.25-(OH)2 VitD indicated normal values. The bone formation marker, total procollagen type I N-peptide (PINP), was 62.1 nmol BCE/L (17.1–64.7 nmol BCE/L), within the normal range, whereas the bone resorption marker, serum N-terminal cross-linking telopeptide of type I collagen (NTX), was elevated at 20.3 nmol BCE/L (7.5–16.5 nmol BCE/L). She was found to have severe osteoporosis.

For the treatment of osteoporosis, injection of denosumab, an anti-receptor activator of nuclear factor kappa-B ligand (RANKL) monoclonal antibody (mAb), was started subcutaneously every half a year from age 36 years. After 1 year, the serum NTX normalized to 8.8 BCE/L, and total PINP maintained in the normal range (20.4 BCE/L). At age 41 years, the bone density in L2–4 and the femoral neck had improved to a T-score of −4.1 SD. Pathological fractures have no longer occurred since the initiation of the treatment with RANKL mAb against bone resorption.

## Discussion

POLR3-related leukodystrophy is characterized by the triad of hypomyelination, hypodontia, and hypogonadotropic hypogonadism and, hence, is often referred to as 4H leukodystrophy. The phenotype of our patient was compatible with 4H leukodystrophy, and compound heterozygous *POLR3A* mutations were genetically confirmed.

A major clinical problem, in this case, was recurrent pathological fractures that occurred during attacks of generalized tonic seizures. Epilepsy is reported in 19–27.9% of POLR3-related leukodystrophy and, when it is present, is typically easy to control (5, 7). In a minority of cases, several antiepileptic drugs are required to achieve seizure control (8). Non-traumatic fractures due to seizures themselves, especially caused by generalized tonic–clonic seizures, have been reported (9). If muscle contraction during a tonic–clonic seizure is strong enough to exceed bone strength, bone fractures could result (10). It is known that antiepileptic drugs such as carbamazepine that induces CYP450 and prolonged immobility can reduce the bone mineral density and promote the severity of osteoporosis (11). This patient also has these conditions. However, we consider that hypogonadotropic hypogonadism plays a key role in severe osteoporosis, as discussed later.

In POLR3-related leukodystrophy, estrogen secretion is insufficient due to gonadotropin deficit, and primary amenorrhea with delayed puberty is reported to occur in 81% of POLR3A-related leukodystrophy (7). Our patient was also amenorrheic and had hypogonadotropic hypogonadism confirmed by undetectable levels of luteinizing hormone, follicle-stimulating hormone, and estradiol. A patient with POLR3-related leukodystrophy who attempted to become pregnant failed to respond to gonadotropin-releasing hormone pulse therapy, but subcutaneous gonadotropin therapy was successful for normal follicular growth, suggesting that hormonal abnormalities are of pituitary origin (12). However, the detailed mechanism underlying insufficient secretion of pituitary hormones in POLR3-related leukodystrophy remains unclear.

Of note, pronounced osteoporosis and repeated pathologic fractures observed in our patient have never been reported in POLR3-related leukodystrophy. We speculate that estrogen insufficiency after hypogonadotropinism in 4H leukodystrophy may be a cause of decreased bone density and early-adult onset osteoporosis. Estrogen plays multifaceted roles in bone metabolism, and reduced estrogen secretion, especially in postmenopausal women, leads to the progression of osteoporosis and increases the risk of fractures (13). Estrogen binds to its receptors and induces apoptosis in osteoclasts while conversely functioning to inhibit apoptosis in osteoblasts (14). Estrogen insufficiency also promotes bone resorption through upregulation of pro-resorptive cytokines such as interleukin-1 and tumor necrosis factor-α in the bone marrow. These cytokines increase RANKL, a key cytokine in promoting maturation and activity of osteoclasts.

Idiopathic hypogonadotropic hypogonadism, or congenital hypogonadotropic hypogonadism, including Kallmann syndrome characterized by olfactory dysfunction, exhibits no pubertal surges in gonadotropins and sex hormones and suffers from osteoporosis (15). Female idiopathic hypogonadotropic hypogonadism patients show osteopenia and delayed bone age, and early-stage hormone replacement therapy along with regular bone evaluations was essential to prevent the progressive decrease of bone density (16).

In addition to estrogen deficiency due to hypogonadotropic hypogonadism, other factors, including prolonged immobility and poor physical conditions such as underweight and malnutrition, could also contribute to the progression of osteoporosis (17). Furthermore, the effects of antiepileptic drugs on bone metabolism should be considered. POLR3-related leukodystrophy can cause epilepsy, so patients with this disorder are often under long-term antiepileptic therapy, such as our patient. Antiepileptic drugs can change bone metabolism and lead to osteoporosis by decreasing the serum vitamin D concentration and calcium absorption (9). Alternatively, the dysfunction of RNA poly III in osteocytes itself might induce the development of osteoporosis in Poll III-related leukodystrophy.

As for the treatment of osteoporosis, denosumab is an anti-RANKL monoclonal antibody that is used as a bone resorption inhibitor to treat osteoporosis (18). After the administration of denosumab, the level of bone resorption markers decreases in a short time, and improvement of bone metabolism is generally observed (19). Actually, our patient demonstrated the restoration of bone density together with the recovery in bone resorption markers and has shown no further recurrences of pathological fracture. Thus, our case indicates that inhibition of RANKL-mediated signaling can be beneficial to prevent the progression of osteoporosis in POLR3-related leukodystrophy.

Although hypogonadotropic hypogonadism is strongly indicated in this patient, it would be necessary to perform gonadotropin-releasing hormone stimulation tests for definite diagnosis of hypogonadotropic hypogonadism, especially before initiating hormone replacement therapy (HRT). Several considerations were argued against HRT in this patient. The patient was already older than 30 years, bedridden, and severely impaired neurologically, and hence, HRT was considered to have little benefit of her activities of daily living. We are also concerned about thrombosis as a common HRT adverse effect, particularly in such a bedridden patient (20).

In POLR3-related leukodystrophy, endocrine abnormalities are underestimated (5), and we should also be aware that early-adult onset osteoporosis could occur, and resulting pathological fractures could be a serious complication that limits patient's activities of daily living. Early evaluation and prophylactic intervention for osteoporosis, including sufficient nutrition, weight-bearing (standing board), and anti-osteoporosis medications, including vitamin D and calcium, should be considered immediately after the diagnosis of POLR3-related leukodystrophy (21). Denosumab could have a beneficial effect as well when estrogen deficiency-induced abnormalities in bone metabolism play a key role in progressive osteoporosis.

## Conclusion

Osteoporosis should be recognized as a potential but serious complication of POLR3-related leukodystrophy. It may be feasible to prevent pathologic fractures by intensive osteoporosis therapy after endocrinological examinations and evaluation of bone metabolism.

## Data Availability Statement

The datasets generated for this study can be found in online repositories. The names of the repository/repositories and accession number(s) can be found in the article/supplementary material.

## Ethics Statement

Written informed consent was obtained from the minors' next of kin for the publication of any potentially identifiable images or data included in this article.

## Author Contributions

SF and YI conceptualized and designed the study. SF wrote the first draft of the manuscript. MKu, HD, NM, and FT contributed to data analysis and assisted in the preparation of the manuscript. SF, TI, KH, NK, AO, MKa, and YI participated in the clinical management and revised the manuscript. MKa, FT, and YI critically revised the final manuscript draft. All authors approved the final version of the manuscript, and agree to be accountable for all aspects of this work.

## Conflict of Interest

NK was employed by the company Fuji Xerox Co., Ltd. The remaining authors declare that the research was conducted in the absence of any commercial or financial relationships that could be construed as a potential conflict of interest.
